# Seroprevalence of HPV serotypes 6, 11, 16 and 18 in unvaccinated children from Mexico City

**DOI:** 10.1017/S0950268819001341

**Published:** 2019-08-30

**Authors:** Reyna Lizette Pacheco-Domínguez, Ramón A. Durazo-Arvizu, Angélica López-Hernández, Jesica Figueroa-Padilla, Julia Berenice Ramírez-González, Malaquías López-Cervantes

**Affiliations:** 1 The National Autonomous University of Mexico, Faculty of Medicine, Research Center on Population and Health Policies, Mexico City, Mexico; 2 Department of Public Health, Loyola University Stritch School of Medicine, Mexico City, Mexico

**Keywords:** Children, HPV, Mexico, preadolescents

## Abstract

Data regarding humoral immunity against HPV infection are scarce. Most analyses focus on the identification of viruses on mucous membranes and primarily refer to women of reproductive age. The aim of this work was to estimate the seroprevalence of antibodies against HPV serotypes 6, 11, 16 and 18 among unvaccinated boys living in Mexico City. A cross-sectional study of 257 male students from 48 public primary schools in Mexico City, whose ages fluctuated between 9 and 14 years, was carried out. Immunological status was assessed by applying the competitive Luminex Immunoassay of HPV (cLIA). Among the study population, we initially found that 38.52% (*n* = 99) of the children tested positive against one or more of the HPV 6, 11, 16 and/or 18 serotypes. The most commonly found serotype was isolated HPV 18 or in combination with other serotypes (22% and 31%, respectively), followed by HPV 6 with frequencies of 4.7% and 11%, respectively; however, lower frequencies were estimated for HPV 16 (2%; 6%) and isolated HPV 11, 4%. If a second set of cut-off points for seropositivity is applied, the overall prevalence for any serotype is reduced to 15.2%. As it appears that a significant sector of the study population has had basal contact with an HPV serotype, we recommend considering the possibility of vaccination against HPV at earlier ages.

## Introduction

Human papillomavirus (HPV) is associated with skin and anogenital warts, as well as cervical, anogenital and oropharyngeal neoplasms [1, 2]. Modes of transmission include sexual and non-sexual contact, and in some parts of the world [3–7], HPV infection is considered to be the most common sexually transmitted disease. Hence, HPV should not be a common condition among school-age children, who are presumably sexually inactive.

Data regarding HPV infection due to HPV in children are generally scarce [8]. Most analyses focus on the identification of viruses on mucous membranes; primarily among women of reproductive age. Thus, there is little information concerning the distribution of HPV infection among the general population, and especially among infant males. This study aims to describe the frequency of antibodies against the HPV serotypes 6, 11, 16 and 18 in a sample of 9- and 14-year-old children, living in Mexico City.

## Material and methods

This study is part of a clinical trial aimed at assessing the immunogenicity and safety of the quadrivalent HPV vaccine (HPV 6, 11, 16, 18), comparing boys and girls to young women. Two serum samples were obtained for the trial; the first sample was drawn prior to vaccination and the second, 1 month after the second dose in the two-dose vaccination scheme. For the purposes of this study, we used only the first sample; that is the one drawn prior to vaccination. The 257 boys participating in this analysis were fifth-graders, enrolled at 54 public primary schools, located in nine of the 16 municipalities that comprise Mexico City. All of the primary caregivers who participated declared that their children had not received any previous HPV vaccination.

Initially, we obtained parental consent and children participating in the study also agreed. Structured interviews in the form of a standardised questionnaire were applied to procure demographic data and weight and height were also measured using standardised procedures and scales. Finally, 5 ml blood samples were obtained, and one serum aliquot was sent to the FOCUS Central Labs Merck, in California, USA, to be processed using the competitive Luminex Immunoassay. This assay measures virus-like particles that indicate antibody concentrations against HPV 6, HPV 11, HPV16 and HPV18 serotypes [8, 9].

We applied the 41, 24, 34 and 39 mMU/ml cut-off points to establish seropositivity, for HPV6, HPV 11, HPV16 and HPV18, respectively. This first set of values was sent to us by the laboratory once sample processing had been accomplished; at that time, we noticed that these values were higher than the cut-off points cited in other studies published previously (see Appendix 1) [10–19]. Subsequently, and after the laboratory checked the statistical outputs and seropositivity estimates, we were provided with a second set of cut-off points with even higher values that changed the overall picture (the second set of values are: 50, 29, 41 and 59 mMU/ml for HPV6, HPV 11, HPV16 and HPV18, respectively).

Hence, Table 2 presents estimates based on the first set of values sent by the Merck Central Laboratory in the first panel; then, the second set results issued by the Merck Laboratory after they had assessed the initial seropositivity estimates is presented in the second panel and panels 3 and 4 present the prevalence estimates based on cut-off points used in the previously published papers.

We also estimated geometric means for the antibody titres, along with their corresponding 95% confidence intervals (Table 1). Finally, we present results for each one of the viral strains, as well as all their possible combinations. All analyses were performed after imputing values for results below the limit of detection (LOD) with LOD/√2 in order to eliminate zero values. This value corresponds to an estimate of the median of the tail distribution for each of the antibody concentrations against the HPV 6, HPV 11, HPV16 and HPV18 serotypes. All LOD values were below 11.3 mMU/ml and imputation was performed to deal with the problem of results below the LOD in order to better estimate true prevalence, instead of treating these cases as missing values. Imputation was made at 14.6%, 28.6%, 28.3% and 3.3% for HPV 6, HPV 11, HPV16 and HPV18 data, respectively. Thus in essence, we considered any results below LOD to be almost zero values, following Durazo and Sempos's argument [20]. Furthermore, the distribution of each of these antibody concentrations was estimated via kernel density estimators with the Epanechnikov kernel function, after randomly imputing values below the LOD [21].

**Table 1.** Basal HPV GMT's by serotype

		HPV serum titres			
Serotype	*n*	GMT	95% CI	Min	Max
HPV6	257	20.3	(18.7–22.0)	11.3	265.6
HPV11	257	12.5	(11.9–13.1)	11.3	218.8
HPV16	257	13.1	(12.4–13.9)	11.3	177.9
HPV18	257	32.5	(30.7–34.5)	11.3	223.2

## Results

The 257 boys who participated ranged in age from 9 to 14 years; the mean age was 10.4 (95% CI 10.3–10.5) years, and the median age was 10 (IQR 10–11) years.

**Fig. 1.**
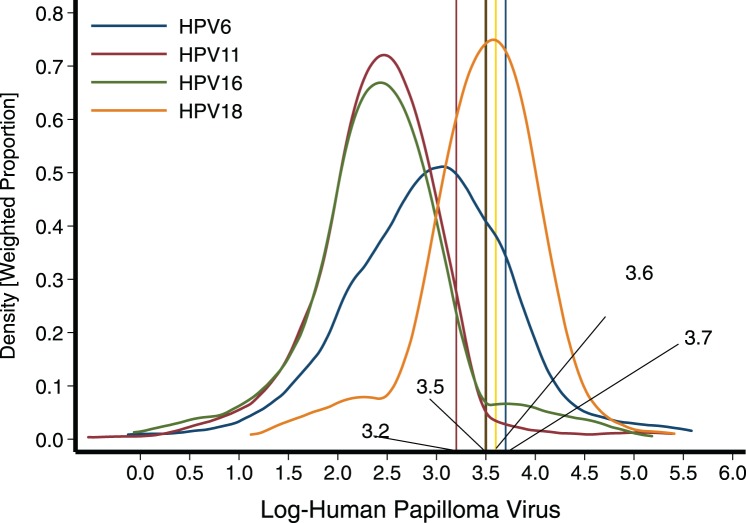
Distribution of antibodies against the four serotypes studied among young boys from Mexico City. To establish seropositivity, we initially applied the cut-off points 41 (log 3.7), 24 (log 3.2), 34 (log 3.5) and 39 (log 3.6) mMU/ml for HPV6, HPV 11, HPV16 and HPV18, respectively.

The geometric means (95% CI, min, max) of the antibodies against HPV 6, 11, 16, 18 were 20.3 mMU/ml (18.7–22.0, 11.3, 265.6), 12.5 mMU/ml (11.9–13.1, 11.3, 218.8), 13.1 mMU/ml (12.4–13.9, 11.3, 177.9) and 32.5 mMU/ml (30.7–34.5, 11.3, 223.2), respectively (Table 1). Figure 1 depicts the distribution for each one of the antibody concentrations against the four serotypes studied.

Overall, using the first set of cut-off points previously mentioned, we estimated that 38.5% of the children presented humoral evidence of contact with any of the following serotypes: HPV 6, 11, 16 or 18. The most common serotype identified was HPV-18, accounting for about a third of the population affected (31.1%), followed by serotype 6 with 10.9% and lower frequencies were observed for serotypes 16 and 11 with 6.2% and 3.9%, respectively (Table 2). In Table 2, we present the seropositivity estimates obtained when applying the second set of cut-off points sent by the Merck Laboratory. Obviously, because these cut-off points are being set at much higher values, seropositivity changed substantially, estimating 7.4, 3.5, 5.1 and 6.2 for HPV 6, 11, 16 and 18, respectively.

**Table 2.** Seropositivity proportions according to different serum cut-offs points (SSCO)

		First SSCO set provided fry MSD			Second SSCO set provided by MSD			Referent 1 SSCO set			Referent 2 SSCO set		
Serotype	*n*	freq	%	95% CI	freq	%	95% CI	freq	%	95% CI	freq	%	95% CI
HPV6	257	28	10.9	(7.6–15.3)	19	7.4	(4.8–11.3)	62	24.1	(19.3–29.7)	139	54.1	(47.9–60.1)
HPV11	257	10	3.9	(2.1–7.0)	9	3.5	(1.9–6.5)	26	10.1	(6.7–14.4)	26	10.1	(6.9–14.4)
HPV16	257	16	62	(3.9–9.9)	13	5.1	(2.9–8.5)	28	10.9	(7.6–15.3)	28	10.9	(7.6–15.3)
HPV18	257	80	31.1	(25.8–37.0)	16	6.2	(39–9.9)	230	89.5	(85.1–92.7)	230	89.5	(85.1–92.7)
Total	257	99	38.5	(32.8–44.6)	39	15.2	(11.3–20.1)	234	91.1	(86.9–93.9)	239	93	(89.2–95.5)

First SSCO set provided by MSD: 41, 24, 34 and 39 mMU/ml for HPV6, HPV 11, HPV16 and HPV18, respectively. Second SSCO set provided by MSD: 50, 29, 41 and 59 mMU/ml for HPV6, HPV 11, HPV16 and HPV18, respectively. Referent 1 SSCO set 30, 16, 20 and 24 mMU/ml for HPV6, HPV 11, HPV 16 and HPV18, respectively (10, 13, 19). Referent 2 SSCO set 20, 16, 20 and 24 mMU/ml for HPV6, HPV 11, HPV16 and HPV18, respectively (11, 15, 16, 17, 18).

These measurements were also apt for identifying single or multiple infections (Table 3). We detected nine out of the 15 possible combinations between the four serotypes. The most frequent category was isolated HPV18 (22.2%), followed by isolated HPV6 (4.7%) and then HPV6/HPV18 (2.7%), with HPV16/18 and isolated HPV16 at 2.0%.

**Table 3.** Combinations of HPV seropositivities

				Original SSCO			2nd SSCO		
Combination of serotypes				Freq	%	95% CI	Freq	%	95% CI
Without basal seropositivity				158					
vph18				57	22.2	(17.5–27.6)	9	3.5	(1.9–6.5)
vph6				12	4.7	(2.7–7.9)	11	4.3	(24–7.5)
vph6	vph18			7	2.7	(1.3–5.5)	0	0.0	NA
vph16	vph18			6	2.3	(1.1–4.9)	0	0.0	NA
vph16				5	2.0	(0.8–4.5)	9	3.5	(1.9–6.5)
vph6	vph11	vph18		4	1.6	(0.6–3.9)	4	1.6	(0.6–3.9)
vph11	vph18			3	1.2	(0.4–3.4)	1	0.4	(0.06–2.2)
vph6	vph11	vph16	vph18	3	1.2	(0.4–3.4)	2	0.8	(0.2–2.8)
vph6	vph16			2	0.8	(0.2–2.8)	1	0.4	(0.06–2.2)
vph11				0	0.0	NA	1	0.4	(0.06–2.2)
vph6	vph11	vph16		0	0.0	NA	1	0.4	(0.06–2.2)
Total				257	100.0		257	100.0	

SSCO, serumseropositivity cut-off.

Antibody reactivity to more than one HPV serotype was less frequent (9.8%), while the presence of a single infection was 28.8%. Unexpectedly, no individual manifested isolated HPV11. Again, when we applied the second set of cut-off points, results changed significantly, and the overall figure of 38.5% of subjects with some evidence of contact with one or more VPH serotypes was reduced to 15.2%.

## Discussion

To our knowledge, this is the first serological survey of HPV infection in boys to be performed in Mexico. The main result was an overall prevalence of 38.5%, indicating contact with any serotype of HPV contained in the quadrivalent vaccine, either alone or in combination [8]. More generally, published epidemiological studies reported prevalence values ranging from 10% to 20%, and low-risk serotype infections (HPV 6 and 11) have been observed more frequently [6]. In our study, the most frequent category of infection was the high-risk serotype HPV 18, followed by HPV 16. Seroprevalence of HPV viruses varies depending on many factors, including geographic location, gender and age [8].

An unexpected line of discussion has to do with the cut-off points being applied to estimate the prevalence. In this regard, the gold standard used to measure antibodies is the technique known as competitive Luminex Immunoassay, but the cut-off points for establishing seropositivity have changed over time. Initially, published studies used cut-off points of 11, 8, 11 and 10 for VPH serotypes 6, 11, 16 and 18, respectively. Later the cut-off points were raised to the values 20, 16, 20 and 24 for the same order of serotypes.

In 2010, Opalka and coworkers reported that the assay had an overall >99% specificity and tested three different methods for establishing cut-off points; for their data. The only dramatic change referred to the serotype 6, which ranged from 0.8 to 114 mMU/ml, but all the rest of the values that could be used for assessing serological status were below 10 mMU/ml, for the serotypes included in the quadrivalent vaccine. Subsequently, they modified the cut-off points, altering them to 30, 16, 20 and 24 for the same order of serotypes [11]. Presumably, these new set of values improved sensitivity.

As previously stated, we received an initial set of cut-off points from the MSD central laboratory, which were 41, 24, 34 and 39 for the same order of serotypes; it is important to note that all these values are higher than those used in the past. Later, we received a second set of values, which were 50, 29, 41 and 59, respectively. We have no explanation as to how these sets of cut-off points were determined by the laboratory. However, considering that the initial set of values were higher than any previously used set of values, we decided to report using the first set provided by the laboratory. Obviously, the estimated prevalence for each serotype diminishes when the second set is applied.

Correspondingly, we consider that the most relevant issue is to reach a cogent agreement in terms of a set of values that are most appropriate for estimating seropositivity, either for vaccinated and/or unvaccinated populations. It is essential to take into account that comparisons of population prevalence across locations or over time also depend on the validity of the cut-off points and the methodology employed for measuring antibody levels.

Vaccination against HPV was initiated in Mexico in 2008 and is restricted to girls who are either in fifth-grade at school or 11 years old, as part of a strategy to prevent cervical cancer [22]. Boys have not been included because the vaccination policy does not embrace prevention of other forms of HPV infection such as genital warts and vaccinating males is not considered an efficient way to prevent cervical cancer in women, especially since the country as a whole is still far from reaching a satisfactory vaccine coverage among girls.

However, the Mexico City government presented an initiative to include males in the public vaccination programme. In this context, the Mexican Ministry of Health suggested implementing a trial focused on the vaccination of males, with the same characteristics of the girls, who comprise the target group of the National Program. Previous studies performed in Mexico included only girls and had the purpose of demonstrating non-inferiority when reducing the vaccination scheme from three to two doses. None of those studies collected serum samples prior to vaccination, assuming that the target group had a negligible prevalence of infection.

Our study was designed to demonstrate non-inferiority of vaccination results among boys using as reference the results of vaccination among young females and that is the subject of another report. However, since we took serum samples prior to vaccination, we were able to estimate the seroprevalences of antibodies against the serotypes included in the quadrivalent HPV vaccine, among the boys in our study population at that very moment.

Findings from our study indicate that an important number of boys may present evidence of contact with HPV, prior to vaccination. It is uncertain whether this infection originated from some type of sexual contact or not, and we attained no information in this regard, as parents had to consent to this type of inquiry. There is evidence suggesting the possible transmission of HPV through asexual routes, either vertical (foetal or perinatal) or horizontal (non-sexual personal contact) [23, 24]; however, the prevalence found in our study could not be explained by considering only asexual transmission. A prospective cohort (HERITAGE study) to evaluate the risk of perinatal transmission of HPV and the persistence of infection in the infant population is currently being carried out in Canada and its results should help to improve understanding of the natural history of HPV infection at an early age [25].

In serological studies with age-matched participants of both genders prior to vaccination, women consistently had similar or higher HPV seroprevalences for all serotypes, suggesting that women may have a higher risk of acquiring HPV infection than men [8]. Thus, it is possible that the prevalence of HPV seropositivity in young Mexican girls may be equal to or even exceed that observed in our boys. Another report lending further support to the last statement showed that Mexican American boys and girls had a seroprevalence of 18.3% for the serotypes HPV 16/18, but the sex-specific seroprevalence was significantly lower among males than females [26].

A study carried out in Germany, with a sample drawn from the general population, observed that the group under 15 years of age had a prevalence of antibodies against serotypes 16 and 18 of <3% for both genders [27]. A study conducted in Brazil reported the presence of high-risk HPV serotypes in girls and boys aged 10–15 years, with a seroprevalence of 9.3% for HPV-16 and 1.9% for HPV-18 [28].

We wish to emphasise that the prevalence of antibodies among the children participating in our study may be considerably higher than values reported elsewhere [27, 28]. Also, our results showed that boys aged 9–14 years had a higher HPV18 seroprevalence than other studies. According to Carter *et al*. serology in isolation tends to underestimate cumulative HPV exposure [29]. Hence, the prevalence observed in our study may even be higher. Hence, our results imply the need to examine the criteria for vaccination at 11 years of age and anticipate the age of vaccination by 1 or 2 years of age, with the hope that the children will be less likely to be infected with HPV. Should it be proved that the serological status of girls, in terms of contact with HPV serotypes, was similar or higher to that reported for boys, this might provide more evidence to establish the best age for vaccination.

It is unclear what the presence of HPV antibodies at early ages in life signifies. For example, we cannot tell whether these boys are at risk of developing diseases such as genital warts or suffering other consequences from HPV infection. We consider our findings very relevant to decisions that ponder the apt time to initiate HPV vaccination during infancy. Finally, this study contributes to understanding the natural history of HPV infection and provides a baseline assessment which will elicit discussions about the possible incorporation of boys into the national HPV vaccination programme.

## Supplementary material

For supplementary material accompanying this paper visit https://doi.org/10.1017/S0950268819001341. click here to view supplementary material
