# Supplementary material for: Seroprevalence of HPV serotypes 6, 11, 16 and 18 in unvaccinated children from Mexico City
Source: Epidemiol Infect. 2019 Aug 30;147:e257. doi: 10.1017/S0950268819001341 (PMC6805765; doi:10.1017/S0950268819001341)
Supplement: Supplementary file 1 [file S0950268819001341sup001.docx]

**Appendix: cut-off points used by other studies previously published**
